# Complications Following Karapandzic Flap Reconstruction of the Lip: A Retrospective Cohort Study

**DOI:** 10.3390/medicina62010012

**Published:** 2025-12-20

**Authors:** Evangelos Kostares, Michael Kostares, Georgia Kostare, Vasiliki Koumaki, Kalliopi Theodoridou, Stefanos Korfias, Georgia Vrioni, Stavros Vassiliou, Konstantinos Kontos, Christos Makos, Athanasios Tsakris, Maria Kantzanou

**Affiliations:** 1 Department of Microbiology, National and Kapodistrian University of Athens, 106 79 Athens, Greece; gkostare@med.uoa.gr (G.K.); atsakris@med.uoa.gr (A.T.); mkatzan@med.uoa.gr (M.K.); 2 Department of Oral and Maxillofacial Surgery, Theagenio Cancer Hospital, 546 39 Thessaloniki, Greece; 3 Department of Otorhinolaryngology—Head and Neck Surgery, “Metaxa” Memorial Anticancer Hospital, 185 37 Piraeus, Greece; michaliskost@med.uoa.gr; 4 Department of Anatomy, National and Kapodistrian University of Athens, 106 79 Athens, Greece; 5 1st Department of Neurosurgery, National and Kapodistrian University of Athens, 106 79 Athens, Greece; 6 Department of Oral and Maxillofacial Surgery, University General Hospital Attikon, School of Medicine, National and Kapodistrian University of Athens, 106 79 Athens, Greece

**Keywords:** Karapandzic flap, squamous cell carcinoma, lip, head and neck neoplasms, microstomia, surgical wound infection, surgical wound dehiscence, cicatrix, hematoma, recurrence, neoplasm metastasis, cohort study

## Abstract

*Background and Objectives*: Squamous cell carcinoma (SCC) of the lip is a common malignancy of the oral and maxillofacial region. Medium-to-large post-excisional defects often require reconstructive techniques that preserve oral competence, function, and facial aesthetics. Despite its broad clinical use, the Karapandzic flap lacks comprehensive evidence describing its postoperative outcomes and associated complications. *Materials and Methods:* This retrospective cohort study evaluated all consecutive patients who underwent lip SCC excision followed by Karapandzic flap reconstruction at a tertiary oncologic center in Greece from 2000 to 2024. Demographic, clinical, pathological, and postoperative data were collected, and complications were categorized as early (wound dehiscence, surgical site infection, hematoma) or late (microstomia, excessive scarring). Statistical analyses included comparative tests and Firth’s logistic regression to explore potential predictors of morbidity. *Results:* A total of 102 patients met the inclusion criteria. Most were male (82.4%) with a median age of 68.8 years, and 94.1% had lower-lip tumors. Early complications occurred in 9.8% of patients and late complications in 17.7%, with microstomia being the most frequent late event (15.7%). Age was the only variable showing a borderline significant association with overall complications. No demographic, clinical, or pathological factor, including lesion morphology, cytological diagnosis, tumor location, or presence of metastasis, demonstrated a statistically significant association with early or late complications. *Conclusions:* Karapandzic flap reconstruction represents a reliable single-stage option for lip SCC defects, demonstrating relatively low complication frequencies and generally favorable functional outcomes. Further comparative studies are warranted to evaluate its performance relative to alternative reconstructive techniques.

## 1. Introduction

Squamous cell carcinoma (SCC) of the lip is the most common malignant neoplasms of the perioral region and predominantly affects the lower lip, reflecting its greater cumulative ultraviolet (UV) exposure [1,2]. Chronic UV radiation, tobacco and alcohol use, fair skin, and immunosuppression are recognized risk factors, while actinic cheilitis serves as an important precursor lesion that can clinically mimic inflammatory conditions of the vermilion [3,4]. A wide range of benign, inflammatory, and infectious disorders, including cheilitis, contact reactions, nutritional deficiencies, and herpes labialis, may alter lip appearance and complicate early recognition of premalignant or malignant changes. Systemic diseases such as atopic dermatitis, Sjögren syndrome, and discoid lupus erythematosus may also manifest with characteristic lip findings, the latter being associated with an increased risk of SCC transformation. Although basal cell carcinoma (BCC) more commonly affects the cutaneous upper lip, SCC is far more prevalent, typically presenting as an indurated ulcer or exophytic lesion [5,6,7].

Early-stage lip SCC is generally associated with a favorable prognosis; however, the risk of regional metastasis remains clinically relevant. A meta-analysis by Bhandari K. et al. [8] reported an occult neck metastasis rate of 17% (95% CI: 10–28%) in patients with primary SCC and a clinically N0 neck, along with a delayed nodal metastasis rate of 8% (95% CI: 1–18%). Similarly, a meta-analysis by Kansara S. et al. [9] found an occult nodal metastasis rate of 10% (95%CI 5–17.5%) in patients with lip SCC and an N0 neck. These data contribute to the ongoing discussion regarding the most appropriate approach to managing the clinically N0 neck in lip SCC, as some surgeons prefer a “wait-and-see” strategy while others advocate for prophylactic neck dissection.

Surgical excision remains the primary treatment for lip SCC, and reconstruction must account for the essential roles of the lips in oral competence, articulation, and facial expression. Depending on defect size, location, and tissue involvement and patient’s medical condition, available reconstructive options include primary closure using V- or W-shaped excisions, Abbe and Estlander flaps, the Bernard–Burow technique, free flaps, and neurovascular advancement flaps such as the Karapandzic flap [5,10,11]. For medium-to-large full-thickness defects, the Karapandzic flap is widely used because it preserves the orbicularis oris muscle, labial arteries, and facial nerve branches, enabling restoration of sphincteric function and sensory continuity [10].

Despite the widespread use of the Karapandzic flap for medium-to-large lip defects, the existing literature provides limited data on its postoperative outcomes and complication profile. Most published studies include small patient cohorts, and important aspects remain understudied. As a result, there is a lack of comprehensive evidence evaluating the clinical performance of this technique. This study addresses this knowledge gap by examining a large 25-year institutional cohort, providing detailed data on postoperative outcomes and complications following Karapandzic flap reconstruction.

The present study aims to provide a comprehensive evaluation of the prevalence, spectrum, and determinants of postoperative complications following surgical management of lip SCC reconstructed using the Karapandzic flap at a tertiary oncologic referral center in Greece.

## 2. Materials and Methods

### 2.1. Study Design and Population

This retrospective cohort study was conducted at the Department of Oral and Maxillofacial Surgery of the “Theagenio” Anticancer Hospital in Thessaloniki, Greece. The study was designed and reported in accordance with the Strengthening the Reporting of Observational Studies in Epidemiology (STROBE) statement for cohort studies [12].

The institutional surgical registry was reviewed to identify all consecutive patients who underwent surgical treatment with Karapandzic flap reconstruction for SCC of the lip between January 2000 and December 2024. All operations were performed by experienced oral and maxillofacial surgeons using standardized surgical techniques and perioperative protocols to ensure procedural consistency. Patients aged 18 years or older with a diagnosis of lip SCC confirmed by histopathological examination of the excised specimen were included in the study. Patients with recurrent SCC of the lip were also included. Additional inclusion criteria required the availability of complete preoperative, intraoperative, and postoperative data, as well as a minimum follow-up period exceeding one month after surgery. Given the nature of the neoplasm (SCC), patients were closely followed in the Out-patient Clinic of the Oral and Maxillofacial Surgery Department, where all necessary clinical examinations were performed and the required radiographic evaluations were arranged in accordance with departmental guidelines. Exclusion criteria included secondary tumors, follow-up shorter than one-month, incomplete medical records, and histological types other than SCC (e.g., melanoma, BCC).

### 2.2. Preoperative Assessment and Surgical Management

All patients underwent comprehensive preoperative evaluation, including documentation of lesion morphology (ulcerative or exophytic) and anatomical site (lower lip, upper lip). In accordance with the institutional protocol, surgical management involved wide local excision of the primary tumor with a clinically (visually assessed) appropriate margin of clinically normal tissue surrounding the lesion to ensure oncologic safety. Reconstruction was performed in all cases using the Karapandzic flap. The Karapandzic flap has been the predominant reconstructive technique for medium- and large-sized lip defects at our institution throughout the 25-year study period, owing to its reliability, preservation of neurovascular structures, and our surgeons’ expertise with this method. For this reason, it was selected as the exclusive reconstructive method for analysis. This neurovascular rotational advancement flap preserves the continuity of the orbicularis oris muscle, labial branches of the facial artery, and branches of the facial nerve, thereby maintaining muscular continuity, oral sphincteric function, and perioral sensation. The design follows the natural nasolabial and mentolabial folds, enabling single-stage restoration of medium to large defects with excellent oral competence, articulation, and aesthetic symmetry.

The choice of reconstructive technique was based on oncologic safety and the requirements for functional and aesthetic restoration. In all cases of SCC, the cervical region was clinically and radiographically evaluated in accordance with the departure’s guidelines to assess potential lymph node involvement. Neck dissection was performed only when clinical examination or imaging studies demonstrated findings suggestive of regional metastasis.

### 2.3. Outcome Measures

The primary outcome was the occurrence of postoperative complications following lip reconstruction. Surgical complications were recorded and verified retrospectively from operative and outpatient records, including wound dehiscence, microstomia, surgical site infection (SSI), hematoma, and excessive scar formation. For the purposes of analysis, complications were further categorized into early and late events. Early complications were defined as adverse events occurring in the immediate postoperative period that could compromise wound stability or require additional intervention, such as SSI, wound dehiscence, or hematoma. Late complications were defined as events arising after wound healing had occurred, typically with functional, clinical or aesthetic impact, including microstomia and hypertrophic scarring. Additionally, oncologic outcomes such as regional (lymph node) and distant metastasis were documented. Secondary variables included patient demographics (age, sex), lesion characteristics (site, morphology) and preoperative evaluation based on the cytological examination of brush samples. Brush exfoliative cytology was performed when requested by the treating surgeon, with specimens collected using a cytobrush, prepared on glass slides, and stained using the Papanicolaou (Pap) method; cytological interpretation followed the standard diagnostic criteria of our pathology department, including evaluation of cellular atypia, keratinization, nuclear-to-cytoplasmic ratio, hyperchromasia, pleomorphism, and dysplastic changes. The cytology-histology agreement was defined by comparing the cytological interpretation of the preoperative brush sample with the definitive postoperative histopathological diagnosis. Brush cytology was considered compatible with malignancy when its interpretation suggested malignant features that aligned with the final histopathological confirmation.

The symptom-to-presentation interval, defined as the patient-reported time elapsed between the initial appearance of the lip lesion and the first hospital visit, and the duration of postoperative follow-up were also evaluated. Microstomia was evaluated during follow-up using objective and subjective criteria. Objective findings included commissural displacement medial to the alar groove or inability to insert a teaspoon into the mouth, while subjective criteria included patient-reported difficulty with eating or maintaining oral hygiene. SSI was defined according to the contemporaneous CDC guidelines and the surgeon’s clinical judgment (e.g., purulent drainage, erythema, warmth, localized pain). Hematoma was considered clinically significant when additional surgical intervention had been required. Wound dehiscence was defined as any postoperative separation of wound edges after closure. Postoperative variables included the final histopathological diagnosis, surgical margin status, the occurrence of postoperative recurrence, and the total duration of follow-up.

### 2.4. Statistical Analysis

Statistical analyses were performed using Stata/BE 19.5 for Mac (StataCorp LLC, College Station, TX, USA). Continuous variables were tested for normality using the Shapiro–Wilk test and visual assessment of histograms and Q-Q plots. Depending on the distributional characteristics, continuous data were to be expressed either as means with standard deviations (SDs) or as medians with interquartile ranges (IQRs). Categorical variables were summarized as absolute counts and percentages. Comparisons between patients with and without postoperative complications that did not meet normality assumptions were analyzed using the Mann–Whitney U test for continuous variables; otherwise, Student’s *t*-test was used. For categorical variables, associations were evaluated using the Pearson χ^2^ test or Fisher’s exact test when expected cell counts were small. Complications were stratified a priori into early (wound dehiscence, SSI, hematoma) and late complications (microstomia, hypertrophic/excessive scarring). All comparative analyses were performed on overall complication and separately for each category to delineate potentially distinct clinical predictors.

To explore potential predictors of postoperative morbidity, Firth’s bias-reduced logistic regression was employed to account for the small sample size and sparse outcome distribution. Univariable Firth logistic models were first fitted for all candidate predictors. Variables with *p* < 0.20 in the univariable analysis and those considered clinically relevant were then evaluated in limited multivariable Firth logistic models, with model complexity constrained by the number of observed events. The results were expressed as odds ratios (ORs) with 95% CIs. All statistical tests were two-sided, and significance was set at *p* < 0.05. No imputation was required as complete data were available for the analyzed variables.

## 3. Results

**Figure 1:**
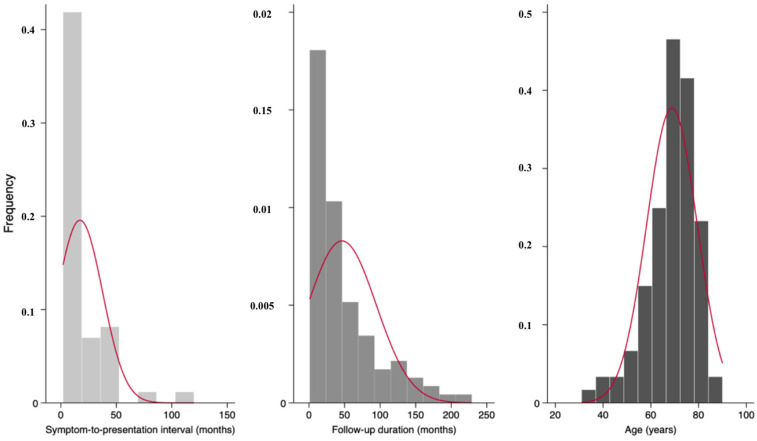
Histograms illustrating the distributions of symptom-to-presentation interval and postoperative follow-up duration.

**Figure 2:**
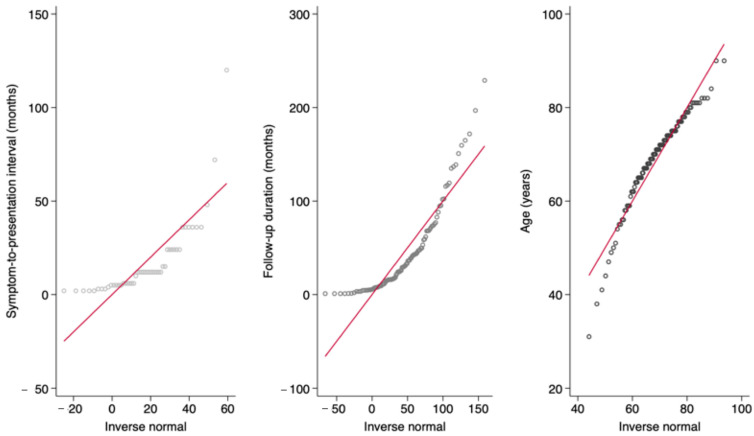
Q-Q plots demonstrating the distributions of symptom-to-presentation interval and postoperative follow-up duration.

Initially, 336 cases of SCC of the lip treated during the study period were identified. After review, 172 patients were excluded because they underwent different reconstructive procedures. An additional 34 cases were excluded due to incomplete medical records and 28 cases had a follow-up period shorter than one month. A total of 102 patients who underwent lip reconstruction using the Karapandzic flap were included. All Karapandzic flaps performed in this cohort were bilateral. The cohort consisted predominantly of men (84 males, 82.4%) with a median age of 71 years (range 31–90). Most tumors originated from the lower lip (94.1%). The cytology-histology agreement was available for 47 patients; among them, 27.7% demonstrated discordance with malignant disease undercalled preoperatively. Regarding complications, early complications (wound dehiscence, SSI, hematoma) occurred in 10 patients (9.8%). Among them, seven cases of wound dehiscence were managed conservatively and healed by secondary intention, while the two patients who developed an SSI were treated with antibiotics along with local wound care and dressing changes. Late complications (microstomia, excessive scarring) were observed in 18 patients (17.65%). Microstomia occurred in 16 patients, and no further surgical intervention was performed, either because it was not indicated or because the patients declined additional surgery. Among the patients who developed recurrence, the median interval from primary surgery to recurrence was 11.9 months, with individual recurrence times of 9.2, 10.9, 12.9 and 20.4 months. Among patients who developed cervical metastasis (12 patients), the median interval from primary surgery to nodal involvement was 8.4 months. During follow-up, two additional patients presented with a palpable cervical swelling without radiologic or cytologic evidence of metastasis; these findings were unrelated to the primary tumor, and the swelling resolved spontaneously. Among patients who developed an SSI, the interval from surgery to infection was 22 days and 6 days, respectively. All surgical margins were negative and considered oncologically acceptable. All continuous variables departed from normality based on the Shapiro–Wilk test (*p* > 0.05) and the evaluation of histograms and Q-Q plots (Figure 1 and Figure 2). All descriptive data are summarized in Table 1.

Postoperative morbidity was not associated with the majority of demographic, clinical, or pathological variables. Age was the only continuous parameter showing a significant difference in patients with overall complications, whereas symptom duration and follow-up length did not differ across groups. Likewise, sex, lesion appearance, recurrence at presentation, preoperative cytological evaluation, tumor location, postoperative recurrence or metastasis, primary surgery with simultaneous neck dissection and cytology-histology agreement showed no significant associations with overall, early, or late complications. In contrast, specific postoperative events demonstrated strong associations: wound dehiscence, microstomia, SSI, and hematoma were linked with increased morbidity in the defined subgroups. Scar formation did not show consistent associations. All detailed statistics for overall, early, and late complications are presented in Table 2.

Univariable Firth logistic regression analyses were performed for overall, early and late postoperative complications. Across all examined variables, no statistically significant associations were identified. Age demonstrated a borderline association in the model for overall complications (*p* = 0.055), whereas its association with early complications was not significant (*p* = 0.112). All other predictors showed non-significant associations with wide confidence intervals in every outcome category. Similarly, none of the categorical clinical variables, including sex, preoperative lesion appearance, preoperative recurrence status, preoperative cytology, tumor site, recurrence, metastasis, metastatic site, primary surgery with simultaneous neck dissection and cytology-histology agreement, demonstrated statistical significance in the models for overall, early, or late complications. The results of all univariable analyses, including odds ratios, confidence intervals, and *p*-values for overall, early, and late complications, are summarized in Table 3.

## 4. Discussion

This retrospective study provides valuable insight into postoperative outcomes following surgical management of lip SCC in Greece. In accordance with previous reports, most tumors involved the lower lip and predominantly affected elderly men, consistent with well-established etiologic factors such as chronic ultraviolet exposure and tobacco use [13,14]. In a retrospective series by Patel et al. [15] involving 50 patients who underwent reconstruction using the Karapandzic flap, SSI occurred in two cases, while combined wound dehiscence and SSI were observed in another two. All patients had preserved oral competence, and none reported microstomia or functional limitations. Likewise, Sumarroca et al. [16] retrospectively evaluated 13 patients treated with the Karapandzic flap and documented no cases of total or partial flap necrosis, four cases of wound dehiscence, and a single case of microstomia. Russo et al. [17] reported that among 13 patients who underwent Karapandzic flap reconstruction, two developed minor wound dehiscence that healed by secondary intention, and all 13 exhibited objective microstomia, while nine also reported subjective microstomia. Furthermore, Teemul et al. [18] reported no requirement for revision surgery and no evidence of disease recurrence at the one-year follow-up among their cohort. Ye et al. [19] retrospectively reviewed 17 patients who underwent reconstruction with a modified bilateral Karapandzic flap and reported two cases of microstomia that required reoperation, no cases of SSI or flap failure and satisfactory oral competence and functional outcomes in all patients.

Importantly, outcomes from more complex reconstructive approaches also support the reliability of techniques incorporating Karapandzic principles. Uglesić et al. [20] reported that a combined bilateral Karapandzic–Abbé/Estlander/Stein flap for subtotal and total lower-lip defects healed uneventfully in all five patients, with no flap necrosis or dehiscence and satisfactory long-term functional results, including preserved oral competence and no drooling. Although all patients exhibited some degree of microstomia, this did not impair oral intake, and aesthetic outcomes were excellent. Their findings show that satisfactory function can be restored even in major defects, though microstomia persists as a notable postoperative concern.

Beyond the incidence of early and late complications reported in previous studies, the present cohort offers one of the largest single-center evaluations of Karapandzic flap outcomes over a 25-year period. Importantly, most complications in our study were minor and managed conservatively, without the need for revision surgery. The predominance of microstomia among late complications aligns with the known physiological consequences of circular advancement and preservation of the orbicularis oris musculature. The lack of statistically significant associations between clinical or pathological predictors and postoperative morbidity is noteworthy. The borderline association between older age and increased overall complications may reflect age-related differences in wound healing capacity or comorbidity burden, although this trend did not reach statistical significance in multivariable testing. This observation nonetheless highlights the importance of individualized perioperative assessment in elderly patients who constitute the majority of those treated for lip SCC.

An additional contribution of this study is the documentation of cytology-histology agreement in a subset of patients. While cytologic brushing is often used as a non-invasive diagnostic adjunct, our finding that 27.7% of preoperative samples underestimated malignant disease underscores the limitations of cytology in lip lesions. This is consistent with the study by Jajodia et al. [21], who also reported notable cytology–histology discordance, including a false-negative rate of 10.9% with conventional cytology and difficulty distinguishing severe dysplasia from invasive carcinoma. Together, these findings emphasize that, while useful as a minimally invasive adjunct, cytology may underrepresent disease severity and should be interpreted cautiously in preoperative decision-making.

This study has several limitations inherent to its retrospective cohort design. Selection bias could have been introduced through the exclusion of patients with incomplete medical records, while sampling bias may limit generalizability since all participants were treated at a single tertiary oncologic center. Attrition bias cannot be entirely excluded, as complete data were not available for all variables across the full cohort. Several preoperative and postoperative parameters were only recorded for subsets of patients, resulting in missing clinical variables that restricted the inclusion of potentially important confounders in the adjusted analyses. Consequently, the statistical analyses for certain outcomes were performed on a reduced number of samples, which may differ systematically from the overall population in ways that cannot be fully assessed. Although no systematic pattern of missingness was identified and the available data did not suggest differential clinical characteristics between patients with complete and incomplete records, the possibility of attrition bias influencing some associations cannot be definitively ruled out. The retrospective nature of the study and the rarity of the examined condition did not allow for an a priori sample size calculation, as the final sample was determined by case availability over the study period. This inevitably limits the statistical power to detect small effect sizes, particularly for rare complications. Therefore, non-significant findings should be interpreted with caution, as a type II error cannot be excluded.

Furthermore, information bias might have arisen from inaccuracies or variability in medical documentation, and some outcomes, such as microstomia or scar formation, may be subject to observer bias. The absence of standardized or objective outcome measures throughout the 25-year study period may have further contributed to variability in assessment. In addition, follow-up protocols were not entirely uniform over time, with differences in timing and documentation across surgeons and eras, which may have affected the detection and classification of postoperative events. The non-randomized nature of the study raises the possibility of confounding by indication, as the choice of surgical technique might have been influenced by factors such as tumor size or patient condition. Additionally, differences in follow-up duration, unavailable or incomplete medical records, variations in surgeons’ experience, and inconsistencies in follow-up assessments or outcome evaluation may have further influenced the findings. Unmeasured confounders, such as patient adherence or postoperative rehabilitation, and the limited granularity of certain clinical variables also represent potential sources of bias.

Additionally, despite the initial plan to perform multivariable regression analysis, the relatively small number of outcome events for both early and late complications limited the feasibility of constructing a stable fully adjusted model, even with the use of Firth penalization. For this reason, the analysis was ultimately restricted to univariable penalized logistic regression to minimize the risk of overfitting and unstable estimates. Therefore, the potential effect of residual confounding cannot be entirely excluded and should be taken into account when interpreting the findings. The wide confidence intervals observed for several odds ratios reflect the limited number of outcome events and the resulting data sparsity, which reduced the precision of some effect estimates despite the use of Firth penalization. This should be considered when interpreting the magnitude of the reported associations. In addition, although clinically relevant confounders were considered during the analysis, the low events-per-variable ratio constrained their simultaneous inclusion in regression models, and residual confounding cannot be excluded.

A further limitation of this study is the absence of TNM staging data. Because the cohort spans 25 years (2000–2024), during which, the AJCC TNM classification underwent several major revisions (6th, 7th, and 8th editions), staging information was not directly comparable across time. Incorporating heterogeneous staging systems would have introduced bias; therefore, TNM staging was not included in the analysis.

Another limitation is the difficulty in making direct quantitative comparisons between our complication rates and those reported in the existing literature. Most published studies on Karapandzic flap reconstruction include very small cohorts and lack standardized or objective criteria, particularly for outcomes such as microstomia, resulting in substantial variability in reported rates. These methodological inconsistencies limit the interpretability of numerical comparison and highlight the need for larger studies with uniform outcome definitions. As this was a retrospective study, standardized grading of microstomia severity and detailed functional scoring were not consistently documented in the medical records and could not be retrospectively applied. Finally, the retrospective design restricted the ability to control exposure variables prospectively or ensure uniform documentation of complications [22,23,24].

## 5. Conclusions

Karapandzic flap reconstruction provides a reliable and function-preserving option for medium-to-large lip defects following SCC excision. In this 25-year single-center cohort, complication rates were low and primarily minor, with microstomia representing the most common late event. Further comparative studies are warranted to clarify its performance relative to alternative reconstructive techniques.

## Figures and Tables

**Table 1:** Demographic and clinical characteristics of the study population (*n* = 102).

Variable	*n* (%)/Median (IQR)
Age (years)	68.8 (61–77)
Sex	
Male	84 (82.4%)
Female	18 (17.6%)
Preoperative clinical presentation	
Ulcerative lesion	61 (59.8%)
Exophytic lesion	41 (40.2%)
Preoperative diagnosis	
Unclear	5 (10.7%)
Negative	8 (17%)
Malignant	34 (72.3%)
Preoperative cytology-histology agreement	
Yes	34 (72.3%)
No	13 (27.7%)
Tumor site	
Lower lip	96 (94.1%)
Upper lip	6 (5.9%)
Tumor presentation at surgery	
Primary SCC	99 (97.1%)
Recurrent SCC	3 (2.9%)
Neck dissection	
No	97 (95.1%)
Yes	5 (4.9%)
Symptoms to presentation interval (month)	12 (6–24)
Follow-up duration (month)	30.8 (11.0–68.3)
Recurrence during follow-up	
No	98 (96.1%)
Yes	4 (3.9%)
Metastasis during follow-up	
Lymph node	12 (11.76%)
Distant (lung)	1 (0.98%)
Postoperative complications	28 (27.45%)
Early complications	10 (9.8%)
Wound dehiscence	7 (6.86%)
SSI	2 (1.96%)
Hematoma	1 (0.98%)
Late complications	18 (17.65%)
Microstomia	16 (15.69%)
Scar formation	2 (1.96%)

**Table 2:** Associations between clinical, pathological, and postoperative variables and overall, early, and late complications.

Variable	Complications					
	Overall		Early		Late	
	z/χ^2^	*p*-Value	z/χ^2^	*p*-Value	z/χ^2^	*p*-Value
Demographics						
Age (years)	2.02	0.043	1.35	0.1754	1.3	0.259
Sex	0.90	0.344	0.62	0.431	0.64	0.423
Preoperative assessment						
Symptom duration	−0.24	0.810	0.21	0.835	−0.22	0.826
Lesion morphology	1.40	0.238	1.06	0.304	0.87	0.350
Recurrent presentation	0.86	0.355	0.28	0.596	0.55	0.460
Preoperative diagnosis	1.07	0.586	0.63	0.729	0.23	0.892
Preoperative cytology-histology agreement	0.54	0.461	0.01	0.901	0.18	0.672
Tumor characteristics						
Tumor site	0.21	0.650	0.77	0.380	1.08	0.299
Neck dissection	0.58	0.445	0.46	0.496	0.02	0.887
Postoperative outcomes						
Local recurrence	0.0005	0.982	0.50	0.478	0.15	0.694
Any metastasis	3.28	0.070	0.31	0.578	1.46	0.227
Metastasis location	0.07	0.782	0.07	0.782	0.07	0.782
Follow-up duration	0.78	0.434	0.73	0.467	0.74	0.456

Continuous variables (age, symptom duration, and follow-up duration) were compared using the Mann–Whitney U test due to non-normal distribution, as confirmed by Shapiro–Wilk testing. All categorical variables were compared using Pearson’s χ^2^ test or Fisher’s exact test when appropriate. Reported statistics correspond to z values for Mann–Whitney U tests and χ^2^ values for categorical comparisons.

**Table 3:** Univariate and multivariate logistic regression analyses of factors associated with postoperative complications, adjusted using Firth’s bias-reduced logistic regression.

Variable	Complications								
	Overall			Early			Late		
	OR	95% CI	*p*-Value	OR	95% CI	*p*-Value	OR	95% CI	*p*-Value
Sex									
Male (ref.)									
Female	0.59	0.16–2.07	0.411	0.61	0.1–3.64	0.586	0.63	0.15–2.64	0.527
Age (year)	0.96	0.92–1.00	0.055	0.95	0.91–1.01	0.112	0.98	0.94–1.02	0.372
Preoperative presentation									
Ulcerative (ref.)									
Exophytic	1.71	0.7–4.12	0.239	1.89	0.56–6.31	0.306	1.62	0.59–4.39	0.347
Recurrent SCC									
No (ref.)									
Yes	0.49	0.02–9.91	0.636	1.39	0.06–30.55	0.835	0.75	0.04–15.61	0.849
Symptom duration (month)	1.01	0.98–1.03	0.954	1.00	0.95–1.05	0.922	1.01	0.98–1.03	0.701
Preoperative diagnosis									
Unclear (ref.)									
Negative	1.91	0.19–18.71	0.579	2.20	0.07–64.9	0.648	1.16	0.11–12.15	0.905
Malignant	0.82	0.11–6.15	0.845	1.23	0.06–27.09	0.899	0.69	0.09–5.23	0.715
Tumor location									
Lower lip (ref.)									
Upper lip	1.65	0.33–8.24	0.546	0.58	0.03–10.84	0.71	2.72	0.53–13.89	0.232
Postoperative recurrence									
No (ref.)									
Yes	1.24	0.17–8.81	0.833	0.85	0.04–16.74	0.912	2.00	0.28–14.47	0.494
Postoperative metastasis									
No (ref.)									
Yes	0.26	0.04–1.45	0.123	0.77	0.13–4.62	0.769	0.42	0.07–2.42	0.33
Neck dissection									
No (ref.)									
Yes	2.15	0.4–11.57	0.376	2.78	0.39–19.63	0.306	1.54	0.23–10.45	0.662
Preoperative cytology-histology agreement									
Yes (ref.)									
No	1.74	0.44–6.92	0.434	1.08	0.14–8.16	0.941	1.47	0.33–6.42	0.615

Only univariable Firth penalized logistic regression analyses are presented. Although multivariable modeling was planned a priori, the limited number of outcome events did not allow for the construction of a stable fully adjusted model, even with penalization.

## Data Availability

Data are available from the corresponding author upon reasonable request.
